# Cyclic Tensile Strain Controls Cell Shape and Directs Actin Stress Fiber Formation and Focal Adhesion Alignment in Spreading Cells

**DOI:** 10.1371/journal.pone.0077328

**Published:** 2013-10-28

**Authors:** Alexandra M. Greiner, Hao Chen, Joachim P. Spatz, Ralf Kemkemer

**Affiliations:** 1 Department of Cell- and Neurobiology, Karlsruhe Institute of Technology (KIT), Karlsruhe, Germany; 2 Department of New Materials and Biosystems, Max Planck Institute for Intelligent Systems, Stuttgart, Germany; 3 Department of Biophysical Chemistry, University of Heidelberg, Heidelberg, Germany; 4 Department of Applied Chemistry, Reutlingen University, Reutlingen, Germany; Karolinska Institutet, Sweden

## Abstract

The actin cytoskeleton plays a crucial role for the spreading of cells, but is also a key element for the structural integrity and internal tension in cells. In fact, adhesive cells and their actin stress fiber–adhesion system show a remarkable reorganization and adaptation when subjected to external mechanical forces. Less is known about how mechanical forces alter the spreading of cells and the development of the actin–cell-matrix adhesion apparatus. We investigated these processes in fibroblasts, exposed to uniaxial cyclic tensile strain (CTS) and demonstrate that initial cell spreading is stretch-independent while it is directed by the mechanical signals in a later phase. The total temporal spreading characteristic was not changed and cell protrusions are initially formed uniformly around the cells. Analyzing the actin network, we observed that during the first phase the cells developed a circumferential arc-like actin network, not affected by the CTS. In the following orientation phase the cells elongated perpendicular to the stretch direction. This occurred simultaneously with the *de novo* formation of perpendicular mainly ventral actin stress fibers and concurrent realignment of cell-matrix adhesions during their maturation. The stretch-induced perpendicular cell elongation is microtubule-independent but myosin II-dependent. In summary, a CTS-induced cell orientation of spreading cells correlates temporary with the development of the acto-myosin system as well as contact to the underlying substrate by cell-matrix adhesions.

## Introduction

The actin cytoskeleton of a cell is a dynamic, adaptive and functional entity ensuring its structural and mechanical integrity. Actin stress fibers, for example, build by actin, myosin IIa and other cross-linkers generate tension forces on focal adhesions by which they are anchored to the extracellular matrix surrounding the cell [1], [2], [3], [4]. This tension is proposed to be a key element in cell matrix rigidity sensing, and in responses to extracellular forces or geometries [4], [5], [6], [7], [8], [9], [10]. The assembly of actin stress fiber and their structural arrangement inside of cells also depends on the matrix rigidity and external forces [4], [11], [12], [13] and it is suggested that several cellular functions, like the differentiation of stem cells are influenced by the architecture of the cytoskeleton [6], [14], [15]. Therefore, the dynamic formation of the cytoskeleton, especially of the filamentous actin networks including actin stress fibers, is a well-studied phenomenon in cell biology. While most adherent cells have *in vitro* a well-developed actin cytoskeleton with stress fibers, the actin cytoskeleton is reduced to a cortical layer in these cells when suspended in a liquid [16], [17]. Upon contact with an adhesive surface, adhesion-dependent cells start to flatten (spread) within hours.They form adhesive contact sites, actin stress fibers and show tension-dependent changes in cell shape such as the polarization of the cell [17], [18]. This spreading of a cell and the establishing of a well-developed actin cytoskeleton depends on the chemical and physical properties of the cells’ environment. For instance, it is suggested that cells adhering on compliant substrates, spread less and show a more condensed actin stress fiber system than cells on stiffer substrates [10]. However, it is not well understood how external forces affect the assembly of the cytoskeleton during cell spreading.

Cells are ubiquitously subjected to mechanical forces in the body. Besides tension and shear flow, cyclic stretching is one of them. The latter one is caused by the extension of blood vessels and their surrounding tissues due to the pulsative character of the heard beat. Cells such as smooth muscle cells, endothelial cell but also adjacent fibroblast are exposed to these cyclic stretching forces and have to adapt accordingly. When cells divide, they first round up, perform cytokinesis and then have to re-adhere to their growth environment while still pulsative forces are acting on them. Thus, studying the behavior of spreading cells subjected to cyclic stretching forces is of broad interest for physiological and pathological events. The actin cytoskeleton and related cell-matrix adhesion sites in fully adhesive cells are responsive to external forces and adapt their structures accordingly. In some studies, adhesive cells are exposed to uniaxial cyclic tensile strain (CTS) applied to the culture substrates. Cells polarize perpendicular with respect to the applied strain and hence reorganize their actin stress fiber system and their adhesion machinery [4], [12], [13], [19], [20], [21], [22]. The actin cytoskeleton has been shown to be essential in the regulation of this stretch-induced cell polarization and plays, together with focal adhesion sites, a key role in this process [6], [9], [12], [23]. Such a force-induced repolarization of well-spread cells and their cytoskeleton is thought to be an avoidance reaction to external stresses or strains acting on cells [24]. Theoretical modeling suggests that the interaction of the contractile force dipole produced by the actin stress fiber and focal adhesion system with the external mechanical force/stress triggers this behavior [9], [25]. In these considerations, the stress-fiber and adhesion system reorganizes in a less stress or strain-exposed direction in order to maintain an optimum internal tension. Experimentally, it has been demonstrated that the reorganization occurs gradually in a continuous way without noticeable disassembly of the fibers [26], [27]. In contrast, Hayakawa *et al.* reported that actin stress fiber reorientation occurs through disruption and reassembly at an oblique angle to the direction of stretch [28]. Other studies demonstrated the involvement of signaling mechanisms, for example, by the activation of RhoA, which depends on the presence of a stress fiber-adhesion system [12], [29]. However, it remains unclear, if the directed assembly of the stress fibers with respect to a stretch axis requires the presence of an intact cellular stress fiber-adhesion system and related signaling mechanisms. The answer to this question is of interest since a) it would reveal if CTS can control directly the *de novo* formation of an actin-focal adhesion system or b) if – as in fully spread cells – a force sensing (focal adhesion) and tension producing system (acto-myosin machinery) needs first to be well-developed before it can lead to the observed cell reorientation responses [30], [31], [32], [33], [34], [35].

We address these issues by fluorescent and phase contrast live cell imaging of spreading cells that are subjected to uniaxial stretching of varying frequencies. We analyzed cell morphology parameters and observed that the cell adhesive area, orientation, and elongation showed a dependency on the stretching frequency while the cell spreading process *per se* was not significantly influenced. We investigated also whether uniaxial CTS (also referred to as stretch) could possibly influence the dynamics of cell spreading as well as the formation of the internal cell polarization, meaning the assembly of the actin stress fibers/focal adhesion system. We observed that in the initial early phase of cell spreading that stretching leads to round isotropic cell morphology with a homogenous radial distribution of nascent cell-matrix adhesions sites, while intracellular anisotropy is established by a parallel alignment of actin stress fibers with respect to the stretch direction.

## Materials and Methods

### Cell Culture, Pharmacological Substances, Plasmids and Transfection

NIH3T3 (from DSMZ, Braunschweig, Germany) were cultured in Dulbecco’s modified eagle serum (DMEM) (Invitrogen, Karlsruhe, Germany) supplemented with 10% Fetal Calf Serum (FCS) (Invitrogen) at standard cell culture conditions (37°C, 5% CO_2_, high humidity). Concentrations of 3 µM taxol, 3 µM nocodazole, 10 µM blebbistatin (all from Sigma-Aldrich, Munich, Germany) were used as indicated in the according experiments. Adherent cells were, prior to detachment from the cell culture vessel, treated with the according pharmacological substance. The level of the cytoskeletal inhibitors was maintained throughout the experiments. GFP-LifeAct was a kind gift from Michael Sixt (IST, Klosterneuburg, Austria) and mCherry-Vinculin from Christoph Ballestrem, (University of Manchester). Transient transfections were performed with Lipofectamin 2000 (Invitrogen) as recommended by the manufacturer.

### Uniaxial Cyclic Stretching Experiments, Light and Fluorescent Microscopy

Stretching experiments for time-lapse phase contrast movies were performed in complete medium and addition of 1% penicillin-streptomycin (Invitrogen) at standard cell culture conditions (37°C, 5% CO_2_) as described in detail elsewhere [12], [21]. Stretching experiments for the time-lapse fluorescent movies were performed using carbonate-free Ham’s F-12 media with L-glutamine (Sigma), 2% FCS (Invitrogen), 25 mM HEPES (Sigma) and 1% penicillin-streptomycin (Gibco). Few cells (10 cells/mm^2^) were let to adhere to the elastomeric fibronectin-coated (Sigma) poly(dimethylsiloxane) (PDMS; Dow Corning Sylgard, Midland, MI, USA) membranes over night and used as “reference cells” for autofocus-controlled image acquisition. The membranes were then clamped into the stretching device and pre-stretched as explained previously [12], [21]. Subsequently, 100 cells/mm^2^ were freshly seeded on the substrates and the stretching of the membrane was started simultaneously. Parameters for uniaxial cyclic stretching (CTS) were set to the frequency (0.1, 0.5, 1, or 3 Hz) at 8% of linear stretch amplitude as indicated in the figures. The stretching device was mounted on an inverted light microscope (AxioVert 200 M, 10x/0.25Ph1 objective or 20x/0.25Ph1 objective or 40x/0.6Ph2 objective, Zeiss, Jena, Germany) or an upright light microscope (AxioExaminer, W-Plan Apochromat 63x/1.0 VIS-IR water immersion objective, Zeiss) equipped with an AxioCam CCD camera (Zeiss). A self-developed software routine embedded in Image Pro 6.2 (Media Cybernetics, Bethesda, USA) or AxioVision 4.6.3.0 (Zeiss) was used. Images for the time-lapse phase contrast movies were acquired at 60 seconds or 120 seconds intervals over 180 minutes. Images for the fluorescent movies were taken at 120 seconds intervals over 180 minutes.

### Analysis of Cell Orientation, Size, and Elongation

#### Cell orientation

Cell orientation was measured as described previously [12], [13]. An ellipse was fitted to each cell outline. The orientation angle φ, of the long axis of the ellipse with respect to the stretch direction was measured. For static experiments (non-stretched control condition) the x-axis of the images was chosen as the reference direction. The orientation angle φ is transformed into the orientation parameter cos2φ. A value of <cos2φ> = 0 corresponds to cells that are randomly oriented; <cos2φ> = 1 if cells are oriented parallel; and <cos2φ> = −1 if they are perpendicularly oriented with respect to the stretch axis.

#### Cell elongation

The elongation was calculated by analyzing the long, i.e. major axis (A_maj_) and the short, minor axis (A_min_) of the ellipse fitted to the cell outline:
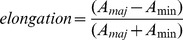
(1)


For *elongation* = 0 the cell shape is a perfect circle, for elongation = 1 the cells a perfect straight.

#### Cell adhesive area

To obtain the cell adhesive area (termed “area”) the cell shape was manually outlined and measured in mm^2^.

### Normalization to Maximum Values

For a direct comparison of the (time-dependent) relationship of cell adhesive area, orientation, and elongation, the data were normalized to their maximum. These normalized time-lapse data were plotted into one graph, as this representation allowed to visualize the relationship of the single parameters to each other over time.

### Detailed Analysis of Actin Stress Fibers, Cell-matrix Adhesions, and Protrusive Activity from Time-lapse Fluorescent Movies

#### Number of actin stress fibers

The number of actin stress fibers per cell was determined by generating a cell mask with ImageJ (http://rsb.info.nih.gov/ij/) and manually counting the number of parallel and perpendicular actin stress fibers with respect to the stretching axis at indicated time points.

#### Orientation of actin stress fibers and focal adhesions

The orientation of actin stress fibers and focal adhesions was calculated with respect to the stretching direction or with respect to the major cell axis. A self-developed software macro embedded in ImageJ was used for analysis as described previously [12].

#### Cell protrusive activity

Protrusions were quantified by dividing the cell in two parts (side: parallel to the stretch axis; end: perpendicular to the stretch axis) and manually counting the cell protrusions in ImageJ. For control (non-stretched) conditions the x-axis of the image was taken as a reference. The dynamics of stretched and non-stretched cells were analyzed at indicated intervals from time-lapse fluorescent data.

### Statistical Analysis

In total 80–120 cells from at least four independent experiments for the time-lapse phase contrast data sets were evaluated. Mean values of cells per indicated time points are presented in the diagrams obtained from the phase contrast data. In total 21 cells at stretching conditions (3 Hz) and 11 cells at control (non-stretched, static) conditions obtained by time-lapse fluorescent imaging were evaluated.

All data were expressed as means ± s.e.m. OriginPro 8G software (OriginLab Cooperation, Northampton, USA) was used for statistical analysis (*t* test, ANOVA) as indicated in the figure legends. Differences were considered as statistically significant when the calculated p value was less than 0.05.

## Results

### The Initial Cell Spreading Phase is Stretch-independent, While Minimum Stretching Forces are Necessary for Cell Orientation in the Later Phase of Cell Spreading

To study the impact of stretching on cell spreading, we subjected freshly adherent fibroblasts to uniaxial cyclic tensile strain (CTS) of different frequencies and a constant amplitude of 8%. We analyzed in detail the impact of stretching on cell adhesive area, cell orientation, and cell elongation.

**Figure 1:**
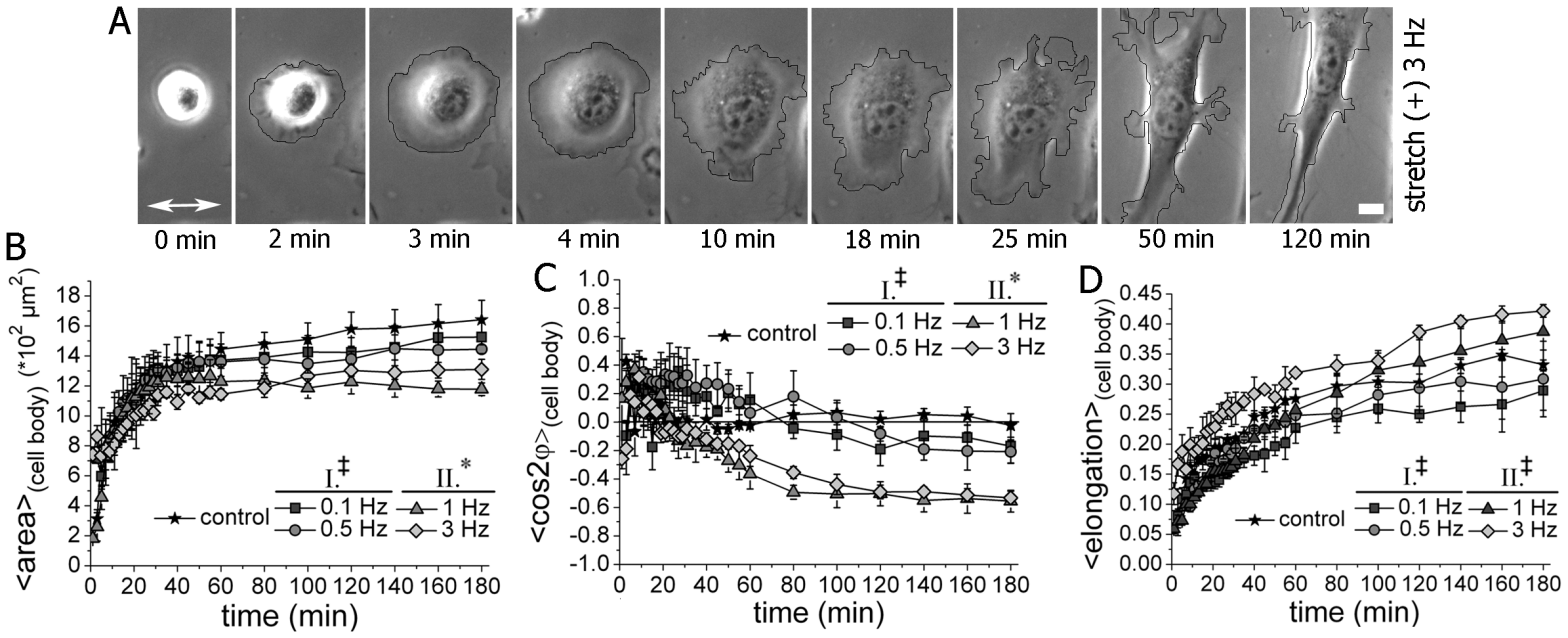
The initial cell spreading phase is stretch-independent while minimum stretching forces are necessary for cell orientation in the later phase of cell spreading. (**A**) NIH3T3 fibroblasts were freshly seeded on fibronectin-coated membranes and cyclically stretched in uniaxial direction (double-headed arrow) with an amplitude of 8% at a frequency of 3 Hz. Cell spreading was monitored via time-lapse phase contrast microscopy. The cell contour of one exemplary cell is outlined in black. (Scale bar: 10 µm) (**B**) Kinetics of the mean cell adhesive area of initially non-adherent NIH3T3 fibroblasts at indicated stretch frequencies (control = non-stretched static condition). Time t = 0 indicates the time point at which cells were seeded onto the substrate. The data set can be divided into two groups depending on the applied frequencies (group I: low frequencies; group II: high frequencies) (ANOVA; group I compared to control: ^‡^p>0.05; group II compared to control: *p<0.05). (**C**) The mean cell orientation at indicated stretch frequencies over time. A mean value of 1 for the orientation parameter <cos2φ> indicates a perfectly-parallel, −1 a perfectly-perpendicular mean cell orientation with respect to the stretch axis. (ANOVA; group I compared to control: ^‡^p>0.05; group II compared to control: *p<0.05) (**D**) Cell elongation over time at indicated conditions. A value of 1 would describe a perfectly round cell, a value of 0 would be a perfect thin line. (ANOVA; group I and II compared to control: ^‡^p>0.05) (control, n = 89 cells; 0.1 Hz, n = 110 cells; 0.05 Hz, n = 80 cells; 1 Hz, n = 120 cells; 3 Hz, n = 117 cells; each from four independent experiments).

**Figure 2:**
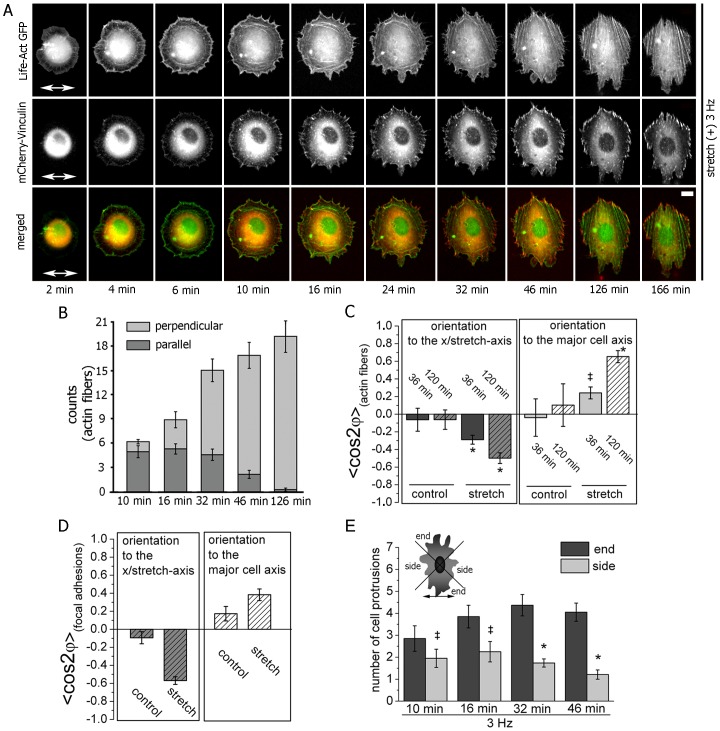
Actin stress fibers reorganization accompanies the stretch-induced cell orientation of isotropic spreading cells. (**A**) Time-lapse fluorescence imaging of a spreading NIH3T3 cell subjected to uniaxial CTS of a frequency of 3 Hz. The cell was double-transfected with Lifeact-GFP and mCherry-Vinculin. A cirumferential actin bundel system and seemingly thickend in parallel to the stretch axis were initially visible after cell seeding. Actin stress fibers realigned into a perpendicular orientation during the time-course of stretching. Cell-matrix adhesion sites emerged homogenously distributed along the cell edges independently of the stretch direction and reoriented during maturation into a perpendicular alignment during stretch application. (Scale bar: 10 µm) (**B**) Count of actin stress fibers confirm that in the initial phase of spreading mainly parallel transversal arc-like actin bundles were detected. The total number of actin stress fibers increased with time and perpendicular ventral actin stress fibers finally dominated. (**C**) Actin stress fiber orientation was analyzed under stretching conditions and static control (non-stretched) conditions. *Left panel:* The actin stress fibers oriented increasingly perpendicular to the stretch axis while they are not aligned under static conditions. *Right panel:* Actin fiber alignment was parallel to the major cell axis under stretch. Even though bipolar cell polarization occurs under control conditions the actin cytoskeleton showed no pronounced orientation with respect to the major cell axis. (T test, 36 min stretch compared to 36 min control: ^‡^p>0.05 for the right panel; *p<0.05 for the left panel. T test, 120 min stretch compared to 120 min control: *p<0.05 for the right and left panel.) (**D**) The orientation of focal adhesions in cells subjected to CTS and under control conditions was evaluated. Focal adhesions oriented perpendicular to the stretch axis (left panel) and thus parallel to the major axis of the perpendicular oriented fibroblasts (right panel). Under non-stretched control conditions no significant preferential alignment of focal adhesion with respect to an arbitrary x-axis or the major cell axis was observed. (**E**) The number and orientation of protrusions was determined under stretch conditions for Life-Act-GFP and mCherry-Vinculin expressing cells. Protrusions that formed at the sides of the cells were defined as parallel; protrusions formed at the end of the cell were assigned as perpendicular to the stretch axis. The number of protrusion was at the beginning equally distributed around a spreading cell with a slightly preferred parallel formation. With increasing time of exposure to CTS the protrusive activity was dominantly at the perpendicular ends of the cell. (T test, side compared to end: ^‡^p>0.05 for 10 min and 16 min; *p<0.05 for 32 min and 46 min) (Stretched condition, n = 21 cells; non-stretched static control conditions, n = 11).

A freshly seeded cell subjected to CTS of 3 Hz made contact with the underlying surface within the first minute (figure 1A and Movie S1; supplemental figure for static non-stretched conditions). The cell area increased and the cell was well-spread after about 20 minutes. The cell spread initially with a round shape (“isotropic”, 15–20 min after cell seeding) before it adopted a bipolar (“anisotropic”, starting about 20 min after cell seeding) cell shape perpendicularly oriented to the direction of stretch (Fig. 1A, 2A, and Movie S1).

Quantification of the cell area over time revealed an exponential characteristic in the increase of this parameter for cells under static and stretching conditions. Cells under static conditions reached their maximum area after 180 minutes. The maximum cell area was not significantly reduced for cells subjected to low stretching frequencies of 0.1 Hz and 0.5 Hz (figure 1B, ^‡^>0.05). However, cells subjected to higher stretching frequencies revealed a significantly decreased maximum cell area compared to cells at static conditions (figure 1B, *p<0.05). This allowed us to distinguish two significantly distinct groups: group I (cells subjected to low stretching frequencies: 0.1 and 0.5 Hz) and group II cells exposed to high stretching frequencies: 1 and 3 Hz) (figure 1B).

Next, we investigated whether cell orientation with respect to the stretch axis is a function of stretching frequency. We determined an orientation parameter for which a value of -1 correspond to perfect perpendicular cell alignment and of 1 to a perfect parallel cell alignment to the stretch axis, while 0 signifies a random cell orientation. We observed that the orientation response of cells exposed to low stretching frequencies was not significantly different compared to cells at static conditions (figure 1C, ^‡^p>0.05). This means that there was no preferred direction for the alignment of the cell body. In contrast, CTS with a frequency of 1 Hz or 3 Hz caused a pronounced perpendicular orientation of the spreading cells with respect to the stretch direction (figure 1C).

We also evaluated the cell elongation. This revealed an increased bipolar cell shape (“polarization”) over time irrespective of the experimental conditions (figure 1D). At low frequencies, the cell elongation was slightly but not significantly reduced compared to cells at static conditions (figure 1D, ^‡^p>0.05). At higher frequencies the cells were more elongated compared to static conditions but the differences were not significant (figure 1D, ^‡^p>0.05).

To analyze the kinetics of cell spreading, the derivative of the cell area (dA/dt) and the standard deviation for the first ten minutes was determined. The control cells showed the fastest spreading during the time period of ten minutes (37.6±3.5 µm^2^/min). Cells subjected to a stretching frequency of 0.1 Hz were not significantly slower (32.9±2.7 µm^2^/min, p>0.01), while cells at 3 Hz had a significantly smaller spreading rate (26.2±3.6 µm^2^/min, p>0.05). This fast spreading period (approx. 20 minutes) was then followed by a period with constant spreading rates (≈6.2±3 µm^2^/min) for all conditions (control and stretching).

To visually demonstrate how cell area, elongation, and orientation are correlated over time, the data were normalized to their maximum ( = 1) and the normalized mean values were plotted into a new graph for one stretching frequency (figure S2, exemplified for 3 Hz). The graph demonstrates that spreading cells subjected to CTS reached their maximum level of adhesive area prior to their maximum level of cell orientation and elongation and the latter two proceed simultaneously.

In summary, the results demonstrate that the cells’ adhesive area, the degree of perpendicular cell alignment with respect to the CTS axis, and the elongation of spreading cells are affected by CTS, but the levels depends on the stretching frequency. The temporal spreading characteristic is not changed upon the application of uniaxial CTS. Spreading occurs regardless of CTS and can be affected, but not stopped by uniaxial CTS.

### Actin Stress Fibers Reformation Accompanies the Stretch-induced Cell Orientation of Isotropic Spreading Cells

We performed fluorescent live cell imaging of transiently double-transfected cells and monitored the actin cytoskeleton and cell-matrix adhesion sites during the process of cell spreading at simultaneous exposure of the cells to CTS (figure 2A and Movie S2 for stretching conditions; figure S3A and Movie S3 for static conditions).

We observed that the cellular shape was nearly radial-symmetric within the first 6–8 minutes of spreading for cells under stretched or static conditions. Within the following period (8–25 minutes), a circumferential, transverse arc-like actin network appeared in both control and stretched cells. In stretched cells, the arc-like actin system seemed to be thicker and more distinct for actin bundles that were parallel to the stretch axis than perpendicular. It seemed that these parallel arc-like actin bundles partially showed a buckling behavior (figure 2A, Movie S2). This spreading phase was then followed by the establishment of a cellular anisotropy, meaning an elongation of the cells. Stretched cells aligned perpendicular with respect to the stretch axis and new thin actin stress fibers assembled *de novo* in a perpendicular direction and thickened (25 to 166 min). The initially present arc-like actin bundles mainly disassembled, while few stress fibers formed along a perpendicular orientation (figure 2A, Movie S2).

At t = 4 min faint, but distinct cell-matrix adhesion sites started to appear radial-symmetrically at the outer cell edge. No obvious differences were observed for cells under static and stretch conditions up to t = 20 min. Subsequently, the cell-matrix adhesion sites quickly matured into elongated focal adhesions and aligned during the maturation process into a perpendicular orientation with respect to the stretch axis. Disassembly of once formed focal adhesions was rarely observed in stretched cells. Remarkably, most focal adhesions formed very early during the spreading process. We observed that about 50% of all actin stress fibers seemed not to be anchored to cell-matrix adhesions and rather merged into other actin bundles, while the other 50% were located at the more ventral side of the cell and were pointing towards adhesion sites (figure 2A, Movie S2 and Figure S3).

We manually quantified the amount of oriented arc-like, dorsal and ventral actin stress fibers (figure 2B). The overall number of actin bundles rose with increasing stretching time. Initially, parallel arc-like actin stress fibers in the cell disappeared whereas perpendicular ventral actin stress fibers finally dominated the internal actin cytoskeletal structure of stretched cells (figure 2B).

Furthermore, we determined the orientation parameter <cos2φ> of the actin stress fibers and focal adhesions under stretch and static conditions. We observed that the internal order of the actin stress fibers and focal adhesions increased over time and both oriented perpendicular to the stretch axis (figure 2C,D). To measure the orientation of the stress fibers and focal adhesion within the cell, we measured their orientation with respect to the major cell axis upon the formation of a bipolar cell (figure 2C, D). On average, actin stress fibers and focal adhesions were only weakly aligned with respect to the major cell axis in cells under static conditions, in contrast to stretching conditions (figure 2C,D). In the latter case, these internal structures were well ordered parallel to the cell axis.

Since the actin stress fiber assembly was obviously influenced by stretch, depending on the cell spreading phase, we also asked the question whether stretching forces have an impact on actin polymerization in cell protrusions. Consequently, we analyzed cell protrusive activity as an indirect measure for oriented actin polymerization in LifeAct and mCherryVinculin-transfected fibroblasts. In the initial 16–20 minutes of cell spreading we observed that the number of cell protrusions perpendicular and parallel to the stretch direction was not significantly different (figure 2E). Formation of significantly more protrusions perpendicular to the stretch axis than to the parallel direction started to appear concomitantly with the cell polarization and actin stress fiber/focal adhesion orientation. However, it was not accompanied by an overall increase in the number of cell protrusions (on average about five to seven protrusion per cell and time point). In cells under static conditions, the protrusion activity was always equally distributed to all cell sides. The overall number of protrusions did not significantly differ between cells under stretch and static conditions (figure 2E; figure S3B).

In summary, cell spreading and attachment under CTS conditions followed a distinct pattern. During the first phase, the cells showed an isotropic spreading with a circumferential arc-like actin network that appeared to be thickened in parallel to the stretch axis, but otherwise with no obvious differences. In the following orientation phase, the cells elongated perpendicular to the stretch direction simultaneously with the *de novo* formation of perpendicular mainly ventral actin stress fibers and the realignment of the pre-existing actin bundles and cell-matrix adhesions during their maturation.

### The Stretch-induced Perpendicular and Polarized Cell Spreading is Microtubule-independent but Myosin II-dependent

We investigate if microtubules and acto-myosin tension are required for the polarized spreading upon stretching. Thus, we interfered with the acto-myosin system or microtubules while spreading cells were stretched with a frequency of 3 Hz. This frequency has been demonstrated to cause a pronounced perpendicular cell orientation (figure 1C).

**Figure 3:**
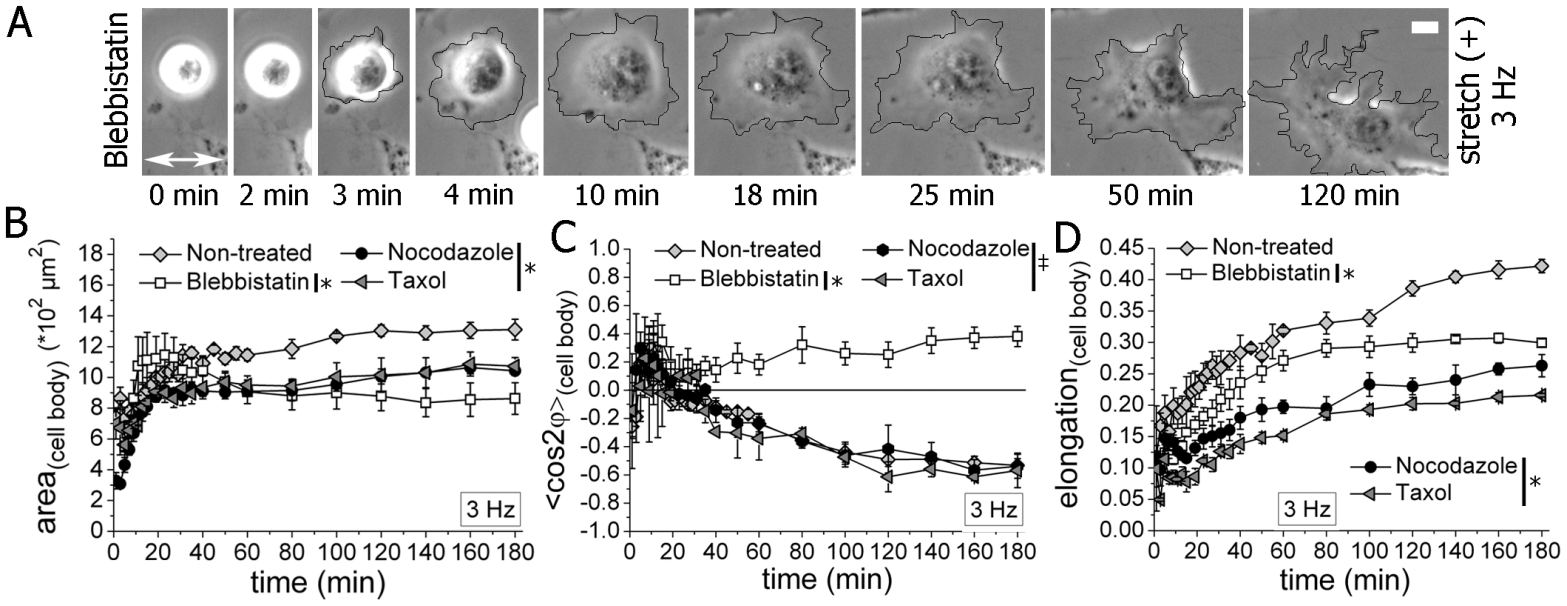
The stretch-induced perpendicular and polarized cell spreading is microtubule-independent but myosin II-dependent. (**A**) Spreading NIH3T3 fibroblasts subjected to uniaxial CTS of 8% amplitude at 3 Hz (double-headed arrow indicates the stretch direction). The cell was pre-treated with blebbistatin. The cell outline of one exemplary blebbistatin-treated cell is given. (Scale bar: 10 µm) (**B**) Kinetics of the mean cell adhesive area of spreading cells at 3 Hz at indicated conditions. The time point t = 0 indicates when the pharmacologically pre-treated cells were seeded onto the substrate. (ANOVA, pharmacologically treated cells compared to non-treated cells: *p<0.05) (**C**) Kinetics of the stretch-induced mean cell orientation of cells treated at 3 Hz with pharmacological substances. Blebbistatin-treated cells tend to orient slightly parallel to the stretch axis while microtubule-disturbed cells aligned perpendicular to the stretch direction. (ANOVA, nocodazole- and taxol-treated cells compared to non-treated cells: *p<0.05; blebbistatin-treated cells compared to non-treated cells: ^‡^p>0.05) (**D**) Time-course of the cell elongation at 3 Hz at indicated conditions. (ANOVA, pharmacologically treated cells compared to non-treated cells: *p<0.05). (nocodazole = disrupts microtubules; taxol = stabilizes microtubules; blebbistatin = inhibits myosin II activity) (Non-treated, n = 117 cells; Blebbistatin, n = 83 cells; Nocodazole, n = 104 cells; Taxol, n = 95 cells; each from four (non-treated sample) to three (pharmacological-treated samples) independent experiments).

Myosin II and microtubules were pharmacologically disturbed, but all treated cells attached to the underlying surface and spread via an intermediate circular shape to their final morphology (amoebic-like for blebbistatin-treated cells and egg-shaped for taxol- and nocodazole-treated cells) independently of stretching (figure 3A; figure S4A).

The cell adhesive area was analyzed for the treated stretched cells. The data were compared to non-treated stretched cells and treated cells under non-stretched, static conditions. We observed that the mean cell area increased rapidly within 20–25 minutes upon cell seeding, similarly for all conditions (figure 3B; figure S4A). Only the maximum area at t = 180 min was significantly reduced for all pharmacologically treated cells (figure 3B).

Cells with inhibited myosin II activity (blebbistatin-treated) significantly aligned their major cell axis parallel to the stretch direction. In contrast, cells treated with microtubule-interfering pharmacological substances over time showed a perpendicular orientation to the stretch axis that did not differ from non-treated stretched cells (figure 3C; figure S4C for cells under static conditions). The cells elongated steadily after cell seeding, independently of the pharmacological substance used and independently of the stretch application. The maximum cell elongation was decreased by about a factor of 1.5 for stretched and non-streched taxol-, nocodazole-, and blebbistatin-treated cells compared to non-treated cells (figure 3D; figure S4D).

Summing up, the disruption or stabilization of microtubules neither influenced cell spreading nor inhibited or reduced the stretch-induced perpendicular alignment process. Interestingly, cells with inhibited myosin II function had not a disturbed cell spreading, but aligned upon uniaxial CTS parallel with respect to the stretch direction and were thus hampered in their stretch-induced perpendicular orientation response.

## Discussion

**Figure 4:**
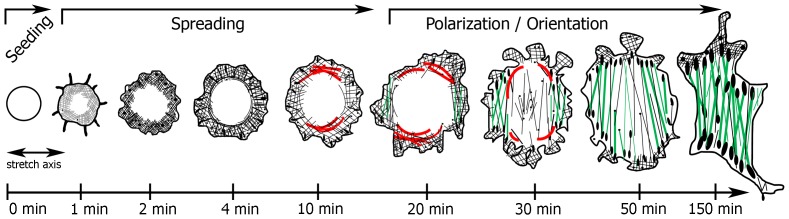
Overview of cell spreading and orientation under cyclic stretch conditions. Spreading of fibroblasts upon CTS application occurs in two phases. In the first phase (“Spreading”) the initial cell attachment is generated. Then a circular lamellipodia and dot-like cell-matrix adhesion sites are visible. The cell reaches its critical adhesive area. In the beginning of the second phase (“Polarization/Orientation”) the cell adhesive area reaches its maximum and cell elongation is initiated. The cell develops with increasing time of stretching pronounced actin stress fibers and cell-matrix adhesions which become with time perpendicularly oriented with respect to the stretch axis. In the spreading phase the formation of actin bundles in parallel to the stretch axis is observed while cell-matrix adhesions emerged homogenously distributed along the cell edges independently of the stretch direction. Cell-matrix adhesions sites reoriented into a perpendicular alignment and the parallel actin fibers realign into a perpendicular orientation and partially disassemble as the stretching force continues to act on the cell.

Our study demonstrates that spreading cells subjected to uniaxial CTS elongate in a perpendicular direction. The spreading process can be divided into two phases (figure 4): In the first phase (“Spreading”; 0 - approx. 20 min), the cell adhesive area increases considerably but the cells do not show a preferential cell body orientation with respect to the stretch axis (figure 4). The cells have a circular lamellipodia and the spatial formation of initial protrusions is not affected by CTS. However, the spreading rate is reduced by increasing stretching frequencies, indicating that the protrusion machinery or respective signaling get disturbed by highly varying substrate forces. With beginning of the second phase (“Polarization/Orientation”), the cell adhesive area reaches its maximum, cell protrusions form preferentially perpendicular to the direction of stretch, and cell elongation is initiated perpendicular to the stretch axis (figure 4). This perpendicular cell orientation coincides with the occurrence of thick perpendicular actin stress fibers. We speculate that a certain integrity of the contractile actin cytoskeleton is necessary for directed actin-polymerization-driven protrusion activity. Similarly, distinct spreading phases have been found and linked to different biochemistry, supporting the idea of a distinct phase of spreading, in which cell contractility and adhesion sites build up and catalyze focal adhesion-dependent pathways [36]. We reveal that the level of the stretch-induced perpendicular cell orientation is dependent on a threshold frequency. As soon as the threshold frequency of 1 Hz is surpassed, the level of perpendicular cell orientation is not further increased with higher frequencies, indicating an “all-or-nothing” reaction. This is in contrast to a study by Jungbauer *et al.* where increasing stretching frequencies lead to a gradual rise of the speed and maximum value of cell orientation [21]. However, in Jungbauer *et al.*, the adherent cells (rat embryonic fibroblasts) are fully spread prior to the experiment. While adherent cells have a well-developed actin network and focal adhesion system, the actin cytoskeleton is reduced to a cortical layer in suspended cells (observation of this study and [17]). Thus, it might be likely that adherent cells with their well-developed actin cytoskeleton and mature cell-matrix adhesion sites are in general more sensitive to changes in stretching frequencies. This idea is also supported by the observation that the cell adhesive area is smaller for freshly seeded cells compared to prior of the stretch experiment well-spread cells [12], [13].

In the first phase of cell attachment and spreading, cells have a circular lamellipodia comprising thin actin fibers radially oriented (figure 4). Small, faint dot-like cell-matrix adhesions appear closely to the lamellum/lamellipodia border. Neither the radial actin bundles nor the small cell-matrix adhesions seem to be influenced by stretching. This is consistent with previous suggestions that early cell-matrix adhesions are either force-independent or require a relatively low tension, which is mainly located in the cell cortex [17], [35], [36], [37], [38]. With continued stretching, thick arc-like actin bundles parallel to the stretch axis appear more prominently. These parallel actin bundles seem to buckle, as also observed under application of compression forces in another study [39]. Cells under static conditions reveal, at that stage of spreading, the assembly of a circumferential actin bundle system (as observed in our studies). Thus, we speculate that the actin bundles parallel to the stretching direction appear more prominent as a result of disturbed structural integrity caused by stretching forces. This disturbance might lead to a loosening of actin-myosin/actin-α-actinin crosslinking [26] in actin bundles resulting in an apparent thickening parallel to the highest strain stretch axis.

With the beginning of the second spreading phase (“Polarization/Orientation”) cells reveal a selective formation of actin bundles perpendicular to the stretch axis. It could be assumed that at this phase the cell is able to sense and thus counteract the stretching forces through a sufficiently developed actin-cell matrix adhesion system and tries to build on internal tension in homeostasis [36], [40], [41]. The mechanical perturbation of the cell thus results in the formation of the actin stress fibers, adhesion sites and cell protrusions perpendicular to the stretch axis, and subsequently results in perpendicular alignment of the whole cell body. It needs to be emphasized that actin stress fibers rather form *de novo* in the perpendicular direction where low mechanical forces are acting on the cell than parallel to the stretch direction. With the development of contractile fiber systems linked to adhesion sites, forces in the extracellular matrix are more effectively transferred to the cell interior than in the early spreading phase. Thus, a mechanical tension in bundles orientated in parallel to the stretching direction, gets periodically disturbed which may lead to a destabilization of these bundles. They do not further mature to stress fibers bundles in this parallel direction. In contrast, fibers exposed perpendicular to the direction of stretch, can stabilize and finally mature to a pronounced stress fiber-adhesion site system in this direction. A similar behavior for stress fiber bundles is suggested by a biochemo-mechanical model for their formation, dissociation, and contraction in fully adhesive cells exposed to uniaxial CTS [42]. Kaunas *et al.* propose that cell reorientation upon periodic stretching as a consequence of disassembly of stress fibers under high strain and a stabilization and formation of stress fibers in orientations that avoid rapid disturbance in their internal tension [43].

New actin stress fibers and cell-matrix adhesion sites assemble relatively fast in a perpendicular direction, as also shown for actin stress fibers in adherent cells by Deibler *et al.* [26]. In general, the acto-myosin machinery is important for the generation of intracellular contractility as well as external traction forces and cellular force-sensing [2], [44], [45], [46]. Previous studies have shown that the actin cytoskeleton, myosin function and thus generated tension were a prerequisite for proper cell spreading [35], [45], [47], [48]. To understand the reorientation process and the necessary prerequisites in more detail we performed experiments modulating the actin network of spreading cells. In the literature, it is described that the inhibition of cell contractility, by blocking myosin II activity or myosin light chain kinase activity, inhibits focal adhesion formation and the force generation of a cell onto the substrate, but does not prevent cell spreading itself [17], [49], [50]. In our study, myosin II-inhibited cells spread well on the substrates and show a preferentially parallel orientation with respect to the stretch axis. This parallel cell alignment might be due to the reduced cell contractility and the lack of an internal tension that is kept at homeostasis. Therefore, the cell cannot actively reorganize in a less strain or stress exposed direction. As a consequence cell structures get passively elongated along the extension axis of the substrate.

Microtubules (MTs) form the rigid shape-determining backbone of a cell and have been shown to be mechano-responsive. They are essential for coordinated cell migration and vesicular transport [51], [52], [53], [54], [55]. By interfering with the MT network of spreading cells, we show that neither MT disruption, nor stabilization has an impact on the spreading process *per se*, as already demonstrated by Cuvelier *et al.* [56]. Additionally, MT dynamics is not essential for a stretch-induced perpendicular cell orientation, indicating that MTs are not necessarily involved in the mechano-response and adhesion process of spreading cells.

Focusing on the temporal evolution of cell spreading steps, we reveal that the process of cell attachment and early spreading is not disturbed for any experimental condition. Thus, attachment and early spreading are independent of the stretching frequency (even though spreading differs in speed depending on the stretch frequency) and independent of an intact acto-myosin and MT system. This is consistent with Cuvelier *et al.* who demonstrat that the dynamics of cell adhesion during the early spreading steps followed a general and conserved behavior independent of cell type, adhesion receptors, and even physical or chemical properties of the substratum [56].

In summary, the formation of a contractile actin cytoskeleton and related adhesion sites in a spreading cell seems to be affected by periodic substrate forces. In contrast, early spreading with mainly protrusion activity and negligible intracellular tension is hardly affected by such forces. The absence of a contractile actin system linked to focal adhesion in the early phase of cell spreading may prevent the effective transfer of forces to the cell interior and related mechano-sensoric signaling [36]. Thus, a CTS-induced cell orientation of spreading cells correlates temporary with the development of the acto-myosin system as well as contact to the underlying substrate by cell-matrix adhesions.

## Supporting Information

Figure S1 **Formation of a major cell axis in spreading cells.** NIH3T3 fibroblasts were freshly seeded on fibronectin-coated membranes under static control (non-stretched) conditions. Cell spreading was monitored via time-lapse phase contrast microscopy. The cell contour is outlined black. Scale bar: 10 µm.
(TIF)

Figure S2 **A threshold frequency lead to dynamic changes in cell area, orientation and elongation and the increase of cell area always precedes the progress of cell orientation and elongation.** Data for the temporal change of cell orientation (<cos2φ>), adhesive area, and elongation under stretching frequency of 3 Hz were normalized to their according maximum and plotted. Cells subjected to cyclic stretching first reach their maximum area followed by cell orientation and elongation with respect to the stretch axis which proceed simultaneously.
(TIF)

Figure S3 **Formation of actin stress fibers, focal adhesions, and protrusions in a spreading cell under non-stretched control conditions.** **(A)** Time-course of a spreading NIH3T3 under static control (non-stretched) conditions. The cell was double-transfected with Lifeact-GFP and mCherry-Vinculin. Actin fibers emerged in a random fashion throughout the cell. Focal adhesions were initially homogenously distributed along the cell edge and were not oriented with respect to the x-axis of the image or the major cell axis, respectively. Scale bar: 10 µm. **(B)** The number and orientation of protrusions was manually counted under control conditions. Protrusions that formed at the sides of the cells (indicated by the small chart in the diagram) were defined as parallel; protrusions formed at the end of the cell were assigned as perpendicular to the x-axis of the image. The number of protrusions per time point per cell was in average between six and eight protrusions and the overall number of protrusions did not increase over time. The protrusions were over the whole time-course equally distributed around the cell.
(TIF)

Figure S4 **Cell spreading of pharmacologically treated cells under uniaxial cyclic stretching and control conditions. (A)** Freshly plated NIH3T3 fibroblasts on fibronectin-coated membranes were uniaxially and cyclically stretched with an amplitude of 8% at a frequency of 3 Hz (double-headed arrow indicates the stretch direction). The cells were either treated with nocodazole or with taxol. Cell spreading was monitored via time-lapse phase contrast microscopy. The cell contour is outlined in black. Each scale bar is 10 µm. **(B)** The mean cell adhesive area of initially non-adherent NIH3T3 fibroblasts at static control (non-stretched) condition over time. The time point zero indicates when the cells were seeded onto the substrate. Cells were treated with different pharmacological substances as indicated. **(C)** Dynamics of the mean cell orientation at non-stretched control conditions. A mean value of 1 for the orientation parameter <cos2φ> indicates a perfectly-parallel, −1 a perfectly-perpendicular mean cell orientation with respect to the x-axis of the image. Cells were treated with different pharmacological substances as indicated. **(D)** Time-course of cell elongation at indicated conditions under control conditions. A value of 1 would be a perfectly spherical cell; a value of 0 would be a perfect thin line. (nocodazole = disrupts microtubules; taxol = stabilizes microtubules; blebbistatin = inhibits myosin II activity).
(TIF)

Movie S1 (MPG)

Movie S2 (AVI)

Movie S3 (AVI)
