# Supplementary figures and images for: Cyclic Tensile Strain Controls Cell Shape and Directs Actin Stress Fiber Formation and Focal Adhesion Alignment in Spreading Cells
Source: PLoS One. 2013 Oct 28;8(10):e77328. doi: 10.1371/journal.pone.0077328 (PMC3810461; doi:10.1371/journal.pone.0077328)

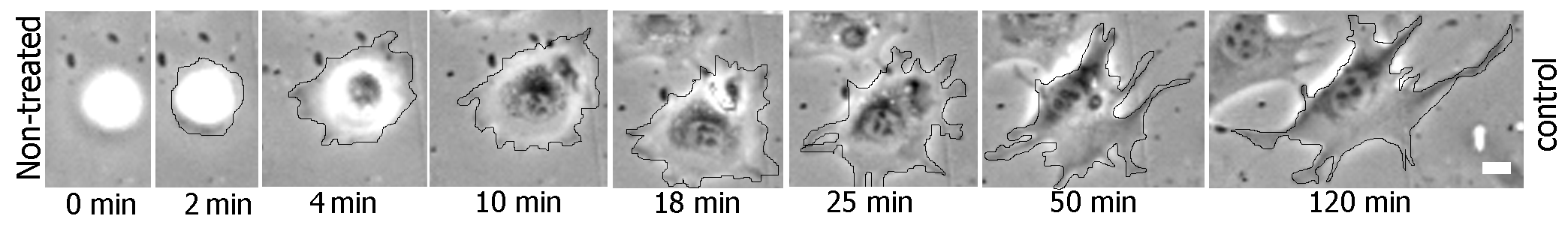

Supplement: Figure S1 — Formation of a major cell axis in spreading cells. NIH3T3 fibroblasts were freshly seeded on fibronectin-coated membranes under static control (non-stretched) conditions. Cell spreading was monitored via time-lapse phase contrast microscopy. The cell contour is outlined black. Scale bar: 10 µm. (TIF) [file pone.0077328.s001.tif]

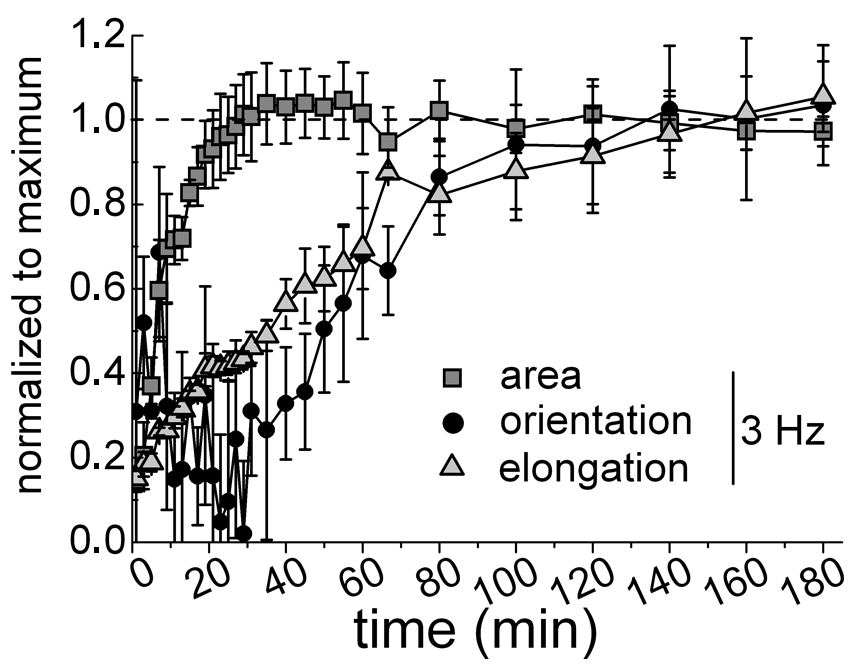

Supplement: Figure S2 — A threshold frequency lead to dynamic changes in cell area, orientation and elongation and the increase of cell area always precedes the progress of cell orientation and elongation. Data for the temporal change of cell orientation (<cos2φ>), adhesive area, and elongation under stretching frequency of 3 Hz were normalized to their according maximum and plotted. Cells subjected to cyclic stretching first reach their maximum area followed by cell orientation and elongation with respect to the stretch axis which proceed simultaneously. (TIF) [file pone.0077328.s002.tif]

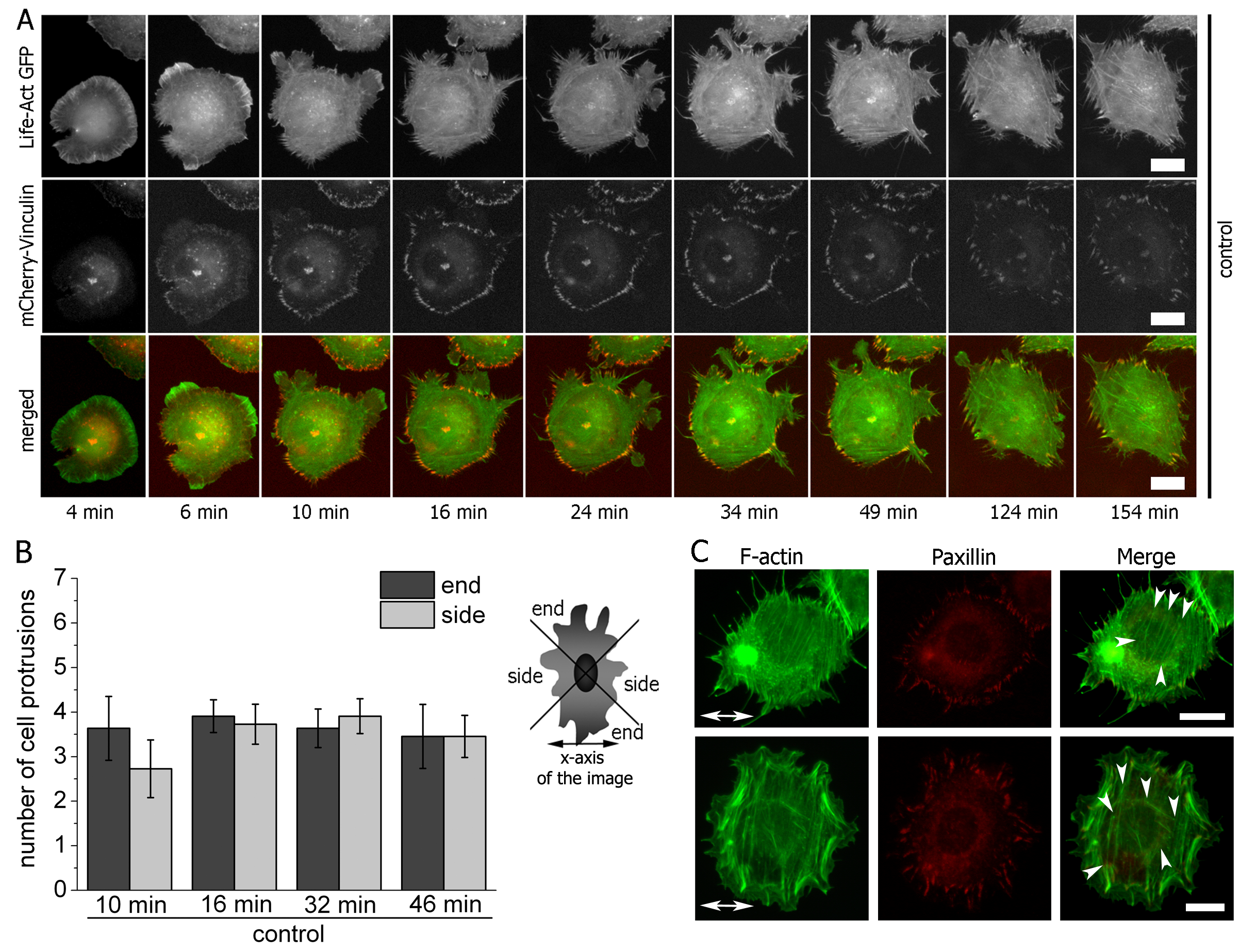

Supplement: Figure S3 — Formation of actin stress fibers, focal adhesions, and protrusions in a spreading cell under non-stretched control conditions. (A) Time-course of a spreading NIH3T3 under static control (non-stretched) conditions. The cell was double-transfected with Lifeact-GFP and mCherry-Vinculin. Actin fibers emerged in a random fashion throughout the cell. Focal adhesions were initially homogenously distributed along the cell edge and were not oriented with respect to the x-axis of the image or the major cell axis, respectively. Scale bar: 10 µm. (B) The number and orientation of protrusions was manually counted under control conditions. Protrusions that formed at the sides of the cells (indicated by the small chart in the diagram) were defined as parallel; protrusions formed at the end of the cell were assigned as perpendicular to the x-axis of the image. The number of protrusions per time point per cell was in average between six and eight protrusions and the overall number of protrusions did not increase over time. The protrusions were over the whole time-course equally distributed around the cell. (TIF) [file pone.0077328.s003.tif]

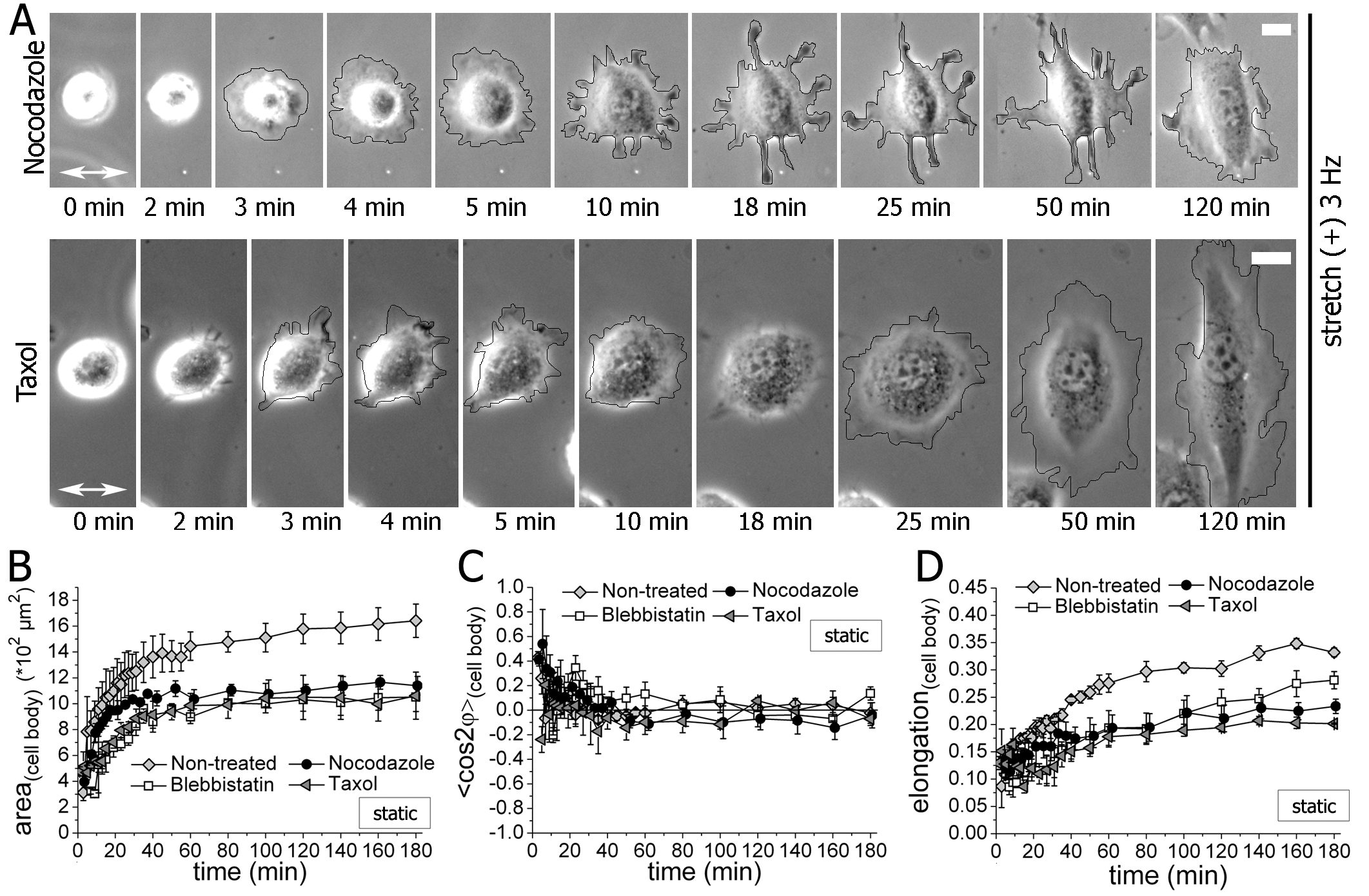

Supplement: Figure S4 — Cell spreading of pharmacologically treated cells under uniaxial cyclic stretching and control conditions. (A) Freshly plated NIH3T3 fibroblasts on fibronectin-coated membranes were uniaxially and cyclically stretched with an amplitude of 8% at a frequency of 3 Hz (double-headed arrow indicates the stretch direction). The cells were either treated with nocodazole or with taxol. Cell spreading was monitored via time-lapse phase contrast microscopy. The cell contour is outlined in black. Each scale bar is 10 µm. (B) The mean cell adhesive area of initially non-adherent NIH3T3 fibroblasts at static control (non-stretched) condition over time. The time point zero indicates when the cells were seeded onto the substrate. Cells were treated with different pharmacological substances as indicated. (C) Dynamics of the mean cell orientation at non-stretched control conditions. A mean value of 1 for the orientation parameter <cos2φ> indicates a perfectly-parallel, −1 a perfectly-perpendicular mean cell orientation with respect to the x-axis of the image. Cells were treated with different pharmacological substances as indicated. (D) Time-course of cell elongation at indicated conditions under control conditions. A value of 1 would be a perfectly spherical cell; a value of 0 would be a perfect thin line. (nocodazole = disrupts microtubules; taxol = stabilizes microtubules; blebbistatin = inhibits myosin II activity). (TIF) [file pone.0077328.s004.tif]
